# The Spending of Half a Million of Money

**Published:** 1907-08-17

**Authors:** 


### HOSPITAL ADMINISTRATION.

### CONSTRUCTION AND ECONOMICS.

### THE SPENDING OF HALF A MILLION OF MONEY.

### THE REBUILDING OF THE LONDON HOSPITAL.

Those who are familiar with the history and development of British hospitals are aware that under the voluntary system it has been the practice, not by combination in the hospital centres, but by the concentrated action of current opinion based upon scientific facts, to reconstruct and often to rebuild the more important hospitals every sixty or eighty years. It is probable that the intervals between one such reconstruction and another for hygienic reasons will be largely reduced in the years that are to come. Indeed, the present system may be entirely revolutionised by the substitution, for ward purposes at any rate, of temporary buildings for permanent structures. Another factor which is likely to enter into the determination of this question of rebuilding is the site. The introduction of motor traction for street and road vehicles of all descriptions must speedily annihilate distance for all practical purposes, and so make it possible, as it has always been desirable, to place every town hospital well outside the city boundaries upon a site wholly isolated from contiguous buildings, and selected only for its hygienic and health-contributing qualities. In this way the ideal of *a Modern Hospital City, outside and distinct from the business and residential city*, which Sir Henry Burdett elaborated during his visit to the United States in 1905, may be much nearer realisation than is generally anticipated. Meanwhile hospital authorities have to face facts as they are, and the result has been that during the last ten or fifteen years the managers of nearly every great metropolitan hospital have had to face the question of rebuilding and to provide enormous funds, which collectively will be found to amount to probably five millions of money, to defray the cost of this work alone.

One powerful lever which has helped the hospital authorities to successfully carry through these necessary schemes of rebuilding has been the institution of the King's Hospital Fund. That fund, in the first years of its distribution, set itself to encourage such necessary works by promising annual contributions of £5,000 a year each to hospitals like Guy's and the London, providing rebuilding was undertaken upon an improved plan which would ultimately result in securing a new, up-to-date, modern hospital. In fact, the London Hospital, which commenced to rebuild in 1897, has completed its building operations in 1907, and they have entailed an expenditure of £491,703. Further, it may fairly be said that the London Hospital has been practically rebuilt throughout.

#### A Herculean Task.

This rebuilding was a herculean task to tackle, and it is not surprising that in 1897 it was decided not to prepare plans for an entirely new hospital, but to build it in sections. Still, we have little doubt that Mr. Sydney Holland, upon whom much of the responsibility and work have devolved, would now be inclined to agree with the view that it would have been better had the first course been taken at the outset. As it happened, during the rebuilding considerable extra expense had to be incurred on account of the committee's decision to close no beds. This involved the provision of temporary buildings of different kinds, which had to be erected and then demolished. Thus temporary wards were put up in the garden at a cost of nearly £4,000, and a temporary out-patient department was built for occupation during the erection of tne permanent buildings. Similarly, a temporary post-mortem department, and a temporary electrical department had to be provided. The most costly single item was the front or central block, which has cost £87,385. Here an entirely new receiving room, new rooms for physicians and surgeons, students and maternity students, with quarters for the steward and new offices for the matron, together with a telephone exchange, a new in-patients' dispensary and new quarters for the Marie Celeste Samaritan Society, have been provided on the ground floor. On the first and second floors are the children's wards, a clinical theatre, clinical class rooms and laboratories, with a servery and registrar's quarters. The third floor is devoted to six new operating theatres for general surgery, anæsthetising and sterilising rooms, with an instrument room and small operation wards. In the basement are a new boiler-house, store rooms, engineers' and electrical shops, and the coal cellars, together with the quarters of the resident laundry-maids and servants. Here, too, provision is made for the filtration of the air for the theatres on the plenum system. In the Grocers' Wing all the wards have been renewed, and each has been fitted with modern windows and lavatory arrangements. This wing also contains the new obstetric wards with operating theatre complete. The work has been efficiently carried out, and everyone who inspects the hospital must be pleased with the vast improvements which have been made.

#### Sections and the Cost.

The vastness of the work will be grasped when we state that reconstruction has been carried out with equal thoroughness and efficiency in the east and west wings, at a cost of £82,770; in the Alexandra wing, including the coroner's court, £12,354; in the pathological department, £19,845; in the isolation block, £29,695. But in addition to the ward accommodation the following new and improved features have been introduced: An entirely new building, known as the Eva Lückes Home for the accommodation of the nursing staff, has been erected at a cost of £50,353; the new laundry, including the price of the site, has cost £35,905; the new boilerhouse,

£3,477; the new house for receiving room officers, £2,283; the temporary quarters for nurses and maternity assistants, £8,052; the rebuilding of the steward's house, £1,228; and in addition £29,823 has been spent upon the furnishing and equipment, including that of the new operation theatres; £13,000 upon the electric lighting throughout the hospital; £2,229 upon garden improvements, including shelters and lodges; £641 upon telephones; and £477 under parliamentary expenses; whilst various smaller improvements under the head of "other works" represent an expenditure of £8,039.

### The Out-patient Department.

Finally, an entirely new out-patient department has been erected at a cost of £82,238. This department has been built on the hospital's own property, an arrangement which necessitated the demolition of 37 houses on the hospital estate, resulting in a serious loss of income and entailing, we understand, an outlay of some £1,200 on the purchase of leases. The out-patient department contains, in addition to the ordinary general surgical and medical departments, large basement store-rooms. It comprises fully equipped departments for aural diseases, for ophthalmic work, for lupus, for dental work, for electrical and *x*-ray treatment, for radiography, and has in addition four operating theatres. The dispensary department has been accommodated in the new out-patient block, and beneath it has been placed a well-appointed pharmaceutical laboratory, fitted up with machinery for making lozenges, tabloids, pills, ointments, and other medicaments. Finally, a special department has been provided and fitted up for medical baths. We have not attempted to give a detailed description of all the manifold improvements which have been introduced, but we hope on a future occasion to attempt a bird's-eye view of the whole institution in its workaday dress as it presents itself to an experienced observer who has attempted to master the details of the work, in all its varying aspects, as it is carried on every day throughout the year.

### Where the Money has come from.

First of all the invested funds have been trenched upon to a considerable extent. On December 31, 1897, these amounted to £233,317, but at the end of 1906 they only represented £120,509, showing that no less a sum than £112,808 had been provided from this source towards the expenses of rebuilding. To this must be added £38,674, *i.e.*, the actual loss accruing to the hospital estate by the sale of some of its property, which brings up the total contributions from invested funds to £151,482. The actual net deficiency to be made good has therefore been £340,221. Of this about £130,000 has come from a few donors of large gifts for specific purposes, and the balance of £210,000 has been raised in the decade by appeals, dinners and other methods which have been actively organised by the chairman, who has displayed great zeal and success in these directions. Finally it should be mentioned that the King's Fund, as already indicated, subscribes £5,000 a year to replace the interest on capital which has been sunk in the reconstruction scheme, a sum it will be seen which represents rather more than three per cent. interest on the £151,483 taken from investments.

